# Toward a Better Testing Paradigm for Developmental Neurotoxicity: OECD Efforts and Regulatory Considerations

**DOI:** 10.3390/biology10020086

**Published:** 2021-01-23

**Authors:** Magdalini Sachana, Timothy J. Shafer, Andrea Terron

**Affiliations:** 1 Organisation for Economic Co-operation and Development (OECD), Environment Health and Safety Division, 2 rue André Pascal, 75775 CEDEX 16 Paris, France; 2 US Environmental Protection Agency, Rapid Assay Development Branch, Biomolecular and Computational Toxicology Division, Center for Computational Toxicology and Exposure MD B105-03, Research Triangle Park, Durham, NC 27711, USA; shafer.tim@epa.gov; 3 European Food Safety Authority, PREV Unit, 43126 Parma, Italy; Andrea.Terron@efsa.europa.eu

**Keywords:** developmental neurotoxicity, in vitro battery, integrated approaches to testing and assessment, adverse outcome pathways

## Abstract

**Simple Summary:**

It is recognized that the current developmental neurotoxicity (DNT) testing paradigm is not fit-for -purpose for the assessment of a large number of chemicals. In the last two decades there have been scientific advances made for evaluating chemical interactions with the developing nervous system that rely on alternative to animal methods. The Organisation for Economic Co-Operation and Development (OECD) provides a forum to develop internationally harmonised guidance to test and assess chemicals for DNT that is primarily based on cellular models. Given the complexity of the developing nervous system and the availability of a number of non-animal methods to address DNT, integration of data from multiple studies is necessary and an OECD framework for organising existing scientific knowledge can be applied as the canvas of this integration. Herein, we provide a brief overview of the OECD DNT project and summarize various achievements of relevance to the project. The review also presents an opportunity to describe considerations for uptake of the DNT non animal methods in a regulatory context.

**Abstract:**

Characterization of potential chemical-induced developmental neurotoxicity (DNT) hazard is considered for risk assessment purposes by many regulatory sectors. However, due to test complexity, difficulty in interpreting results and need of substantial resources, the use of the in vivo DNT test guidelines has been limited and animal data on DNT are scarce. To address challenging endpoints such as DNT, the Organisation for Economic Co-Operation and Development (OECD) chemical safety program has been working lately toward the development of integrated approaches for testing and assessment (IATA) that rely on a combination of multiple layers of data (e.g., in vitro, in silico and non-mammalian in vivo models) that are supported by mechanistic knowledge organized according to the adverse outcome pathway (AOP) framework. In 2017, the OECD convened a dedicated OECD expert group to develop a guidance document on the application and interpretation of data derived from a DNT testing battery that relies on key neurodevelopmental processes and is complemented by zebrafish assays. This review will provide a brief overview of the OECD DNT project and summarize various achievements of relevance to the project. The review also presents an opportunity to describe considerations for uptake of the DNT in an in vitro battery in a regulatory context.

## 1. Introduction

It is well documented that certain chemicals can adversely interact with the development of the nervous system in humans [1]. There are epidemiological studies and systematic reviews available in the scientific literature describing how exposure to chemicals may be associated with an increased risk of some neurodevelopmental disorders, including autism, attention deficit disorder and mental retardation [2,3,4,5]. Although the association between chemical exposure and adverse neurobehavioral and neurodevelopmental effects still remains to be clearly demonstrated, it is expected that ongoing efforts worldwide, such as the Neurosome and the Japan Environment and Children’s Study (https://www.neurosome.eu/, https://www.env.go.jp/chemi/ceh/en/index.html) and new research advancements [6] will help to begin to clarify the possible causal associations between early life environmental chemical exposure and neurological disorders in children. Recognition of the importance of safeguarding the developing nervous system from chemical exposure is evident through the acknowledgment that characterization of developmental neurotoxicity (DNT) hazard potential for chemicals is considered for risk assessment by various regulatory sectors and is embedded in many regional legislations.

Historically, the unique vulnerability of the developing brain to chemicals has been assessed using EPA or OECD Test Guidelines [7,8] that are based on animal models and investigate alterations in neuroanatomical, neurophysiological, neurochemical and neurobehavioral parameters following perinatal chemical exposures [9,10,11]. However, no standard regulatory data requirements for DNT currently exist in any of the chemical regulatory sectors; most commonly, an observational finding related to neurotoxicity detected in other routinely conducted in vivo studies might trigger in vivo DNT testing [12]. This fact, accompanied by substantial resource requirements, test complexity, difficulty in interpreting data and ethical reservations, limits the use of the in vivo DNT test guidelines. This has resulted in scarce DNT toxicological hazard information, which is estimated to be available only for 110–140 [9,13] compounds. With over 350,000 chemicals and mixtures of chemicals registered on the global market [14], more efficient and cost-effective methods are needed for assessing hazard, exposure and, ultimately, risk.

The limited DNT in vivo testing, coupled with the recent paradigm shift in toxicity testing which is based on the development and use of pathway-based testing strategies rather than traditional animal–based methods, triggered interest in the research community that subsequently developed new alternative methodologies (NAMs) for DNT [15,16,17,18,19,20,21]. Indeed, decades of research to understand the impact of various types of chemicals on key neurodevelopmental processes (i.e., progenitor cell proliferation, differentiation into neuronal and glial cells, migration, apoptosis, axonal and dendritic outgrowth, myelination, synapse formation and formation of functional networks) advanced the design and development of phenotypic in vitro assays for testing DNT that were recently reviewed for their readiness and use in the regulatory arena [22,23].

This review paper provides an overview of an international effort to develop guidance on in vitro assays for use in a DNT testing battery under the auspices of the OECD. It highlights the various achievements so far and describes the scientific and experimental basis of the DNT in vitro testing battery that is proposed in the guidance. The review also presents considerations for uptake of the guidance that could facilitate countries and industry to enhance DNT testing of chemicals, focusing on the incorporation of mechanistic understanding and practical examples that are applicable in a regulatory context.

## 2. Overview of the OECD DNT Project

Since 1981, OECD has developed international recognized standards, the OECD Test Guidelines (https://www.oecd.org/chemicalsafety/testing/oecdguidelinesforthetestingofchemicals.htm) that are harmonized toxicity testing methods. The majority of these guidelines that are relevant to human health endpoints are animal-based and are widely used to generate data for consideration in chemical safety evaluations. During the last two decades, the Test Guidelines Programme started to focus on alternative testing approaches based on cellular models in response to the evolving regulatory needs of OECD member countries and rapid scientific developments in the field.

Over the last 15 years, a number of scientific workshops and meetings have raised awareness about the fact that the majority of the chemicals released into the environment, and to which children are potentially exposed, have not been evaluated for DNT hazard [15,16,21]. These international efforts highlighted the need for a new framework that would allow cost- and time-efficient DNT testing [24,25]. Recently, a scientific consensus has emerged that the development of in vitro, in silico and alternative species test methods, and the integration of data derived from these methods, could facilitate the evaluation of chemicals, especially in regard to their potential to disrupt brain development [25,26,27]. To address this need, an OECD project was initiated following a workshop jointly organized by OECD and European Food Safety Authority (EFSA). Participants in this workshop agreed that the proposed DNT in vitro testing battery [22] could be further harmonized in an international acceptance process. This would facilitate its use not only for chemical screening and prioritization but also for hazard characterization [22,25].

The purpose of the OECD project, overseen by an OECD Expert Group on DNT, is to deliver a guidance document that will introduce a framework to facilitate the regulatory use of DNT in vitro data derived from the battery through an integrated approach to testing and assessment (IATA) (http://www.oecd.org/chemicalsafety/risk-assessment/iata-integrated-approaches-to-testing-and-assessment.htm). Although it is not envisioned as a direct replacement of the in vivo Test Guidelines, there are a number of regulatory relevant scenarios for which data from the DNT in vitro test battery could be applied to inform decision-making. The majority of these scenarios will be captured in case studies that will illustrate the applicability of the DNT in vitro battery (IVB) in a regulatory context. Moreover, it is envisioned that the Adverse Outcome Pathway (AOP) framework that was formalized by the OECD in 2012 (https://www.oecd.org/chemicalsafety/testing/projects-adverse-outcome-pathways.htm) will be the basis of organizing data and developing IATA (see Section 4).

**Figure 1:**
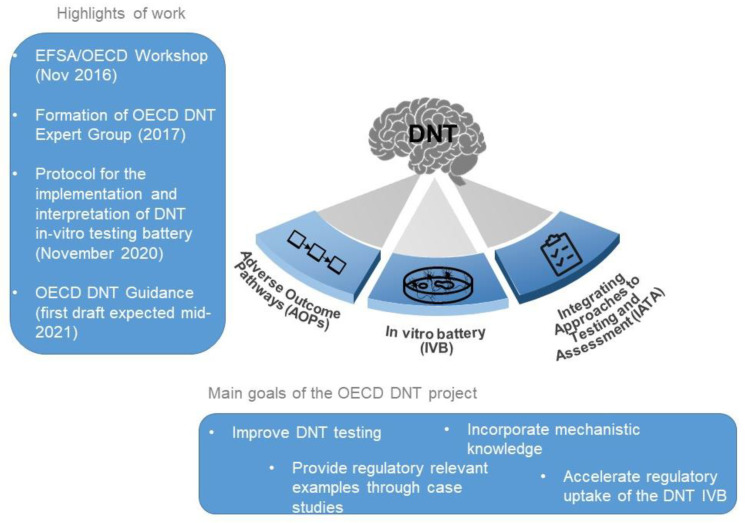
The three pillars of the Organisation for Economic Co-Operation and Development (OECD) developmental neurotoxicity (DNT) project: adverse outcome pathways (AOPs) AOPs, (in vitro battery) IVB, and integrated approaches for testing and assessment (IATA).

The DNT-IVB (described in Section 3) was used to generate extensive experimental data initially by an EFSA-funded research project involving the Universities of Konstanz and Dϋsseldorf, with additional data to be generated by the US Environmental Protection Agency (US EPA). A protocol for the implementation and interpretation of the DNT-IVB has been published [28]. These efforts aim to support the development of an OECD guidance document on the interpretation and use of DNT-IVB data in regulatory decisions that is expected to be finalized in 2021. Figure 1 illustrates the main pillars of the OECD DNT project (i.e., AOPs, IVB and IATA) together with the main highlights and goals of the project, including the long-term objectives to improve DNT testing for chemicals and accelerate the uptake of the upcoming guidance document on DNT, which is expected to be finalized in 2021.

## 3. Scientific and Experimental Basis of the DNT In Vitro Battery (DNT-IVB)

Table 1 provides a list of the assays that are currently included in the battery. It is not intended to provide a detailed description of the assays, but rather a brief summary with references to more detailed descriptions of the assays. This battery of assays was developed around the concept of designing phenotypic testing approaches for key neurodevelopmental processes. This approach was developed through a series of international meetings with scientists, regulators and stakeholders interested in DNT [15,16,17,21,26]. Although approximately 30 or more potential assays have been developed, the ones selected for this battery have been evaluated for their readiness [22] and/or followed the recommendations of Crofton et al., 2011 [21] in their development. In addition, the laboratories that developed the assays in this proposed battery expressed a willingness and had the resources to pursue additional chemical testing with these assays [28]. Thus, it is important to note that an in vitro assay, which is not listed as part of this battery, might still be able to provide useful data for a decision regarding DNT. However, one would need to closely evaluate that assay to determine its validity and reproducibility with known positive and negative control compounds.

### 3.1. Assays of the Proposed Battery

The systematic testing of compounds in a battery of in vitro assays that evaluate key neurodevelopmental events is being done by researchers at three major partner institutions: the US EPA, the University of Konstanz and the University of Düsseldorf. A brief description is provided in the table below for each assay in the battery, including the key neurodevelopmental process being tested, the assay endpoints and additional references further describing the assay.

### 3.2. Processes behind Selection of the Chemicals for Testing

A small (20–30) but not completely overlapping number of chemicals had been tested in each of the assays in the DNT-IVB at the beginning of this project. Thus, the overall goal in selecting chemicals for testing was to maximize the number of chemicals that had been tested across all the assays described in Section 3.1. As a starting point, a list was compiled that included all chemicals tested in the assays as of approximately 2017, when the chemical selection process was started (see Figure 5 in Bal-Price et al. [22]). An expert group of neurotoxicologists met regularly and considered additional compounds from the following sources: those compounds listed as having considerable evidence for in vivo neurotoxicity in Mundy et al., 2015 [15] and/or Aschner et al., 2017 [44]; compounds for which guideline DNT studies were known to exist; compounds that were of interest for IATA development by members of the OECD DNT expert group; putative negative control compounds (primarily from Aschner et al., 2017 [44]; Harrill et al., 2018 [29]) and compounds in the National Toxicology Program’s set of 91 chemicals that had been distributed to multiple labs for DNT testing [45]. This resulted in a list of compounds that contained approximately 310 chemicals. This list was then “cross-checked” with the ToxCast chemical library at the US Environmental Protection Agency to determine which compounds could be obtained through that source and distributed to the labs involved in testing. This eliminated approximately 75 compounds for reasons such as volatility, status as a controlled substance (e.g., cocaine) and regulatory disposal issues (e.g., polychlorinated biphenyls). Of the approximately 240 remaining chemicals, those that had been tested in one or more, but not all, of the assays in Section 3.1 were given high priority for testing in the other assays. Compounds for which there was in vivo evidence for DNT [15,44] and available Test Guideline DNT studies were also given a high priority, followed by those that were of interest for IATA case studies proposed by OECD DNT Expert Group Members, and finally putative negative compounds. This resulted in a list of approximately 120 compounds for testing that were available through ToxCast. Individual plates were made for each assay or group of assays based on how many of the 120 compounds had already been tested in that assay, and the untested compounds were plated and provided to the laboratories. The EFSA-funded researchers at the Universities of Konstanz and Dϋsseldorf have completed their testing and submitted a report to EFSA [28], while data collection for the US EPA assays should be complete by early 2021.

### 3.3. Availability of In Vitro DNT Data

The data from the EFSA-funded project can be found in the report [28] and on the link to that report (https://efsa.onlinelibrary.wiley.com/doi/10.2903/sp.efsa.2020.EN-1938; see “supporting information file: efs31938e-sup-0001-Annex-A.xlsx”). When it is complete, the data from the US EPA laboratory will be made available through the US EPA’s CompTox Chemicals Dashboard (https://comptox.epa.gov/dashboard/chemical_lists/TOXCAST), but this is not expected to be available to the public before mid-2021. Finally, although separate from this project, the National Toxicology Program at the US National Institute for Environmental Health Sciences maintains a DNT-related database (DNT DIVER, https://sandbox.ntp.niehs.nih.gov/neurotox/) that houses data from several of these assays as well as zebrafish behavioral assays.

### 3.4. Usefulness of the Battery

Several regulatory-driven scenarios that illustrate the usefulness of the DNT_IVB are discussed in Section 4.1. In addition, because the battery is based on the disruption of important neurodevelopmental processes, it provides information on potential mechanisms or modalities that a compound is disrupting that cannot be obtained from in vivo guideline studies. As such, it can provide information to help develop DNT-related AOPs, as discussed in greater detail in Section 4.2. Further, such information could also be used to provide scientific rationale for more focused hypothesis-driven in vivo studies when necessary. Ultimately, when implemented in the context of IATAs, the battery will have many potential uses that could inform decision-making relative to DNT.

## 4. Considerations for Uptake of the DNT In Vitro Battery in a Regulatory Context

### 4.1. Case Studies

All stakeholders agreed that a flexible and fit-for-purpose approach is needed to cover the different regulatory needs [27]. It is therefore expected that the OECD guidance currently under development would include guidance on the use of new approach methodologies (NAMs) for regulatory decisions regarding DNT. The guidance will be developed based on problem formulation for different regulatory scenarios, and case studies will help to articulate how to apply the NAMs to these scenarios using an IATA approach. Case studies are therefore expected to cover examples that range from observing apical outcomes in the in vivo study to mechanistic evaluation in assessing DNT in a regulatory context.

Targeted DNT-IVB testing should therefore be geared through a problem formulation approach based on regulatory needs using an IATA framework, with the idea that the endpoints included in the DNT-IVB would fill the gap represented by the lack of mechanistic understanding of DNT. Examples of regulatory scenarios identified by the DNT Expert Group include:

(1) Use of the DNT-IVB for identification/confirmation of biological activity when predictive computational models (including outcome from QSAR analyses) of DNT identify potentially active compounds;
(2) Screening for prioritization of large numbers of chemicals that lack sufficient data on DNT;
(3) Screening of small numbers of class-specific chemicals or mixtures;
(4) Single chemical hazard assessments related to Weight of Evidence (WoE) analysis as part of, e.g., a DNT tiered approach when no DNT data exist, when available in vivo DNT data exist but are inconclusive or when concern arises from new data on alternative species or from the literature.

While scenarios 1 and 2 will cover the needs for potential DNT hazard identification, scenarios 3 and 4 are expected to test the impact of the DNT-IVB on hazard characterization.

Based on these regulatory scenarios, several case studies were proposed by the members of the OECD DNT expert group.

(1) Two IATA case studies on DNT hazard characterization for pesticides, with the inclusion of the DNT-IVB in the AOP-informed IATA approach;
(2) IATA case study on screening DNT hazard identification for organophosphorus flame retardants;
(3) IATA case study on DNT hazard characterization for neonicotinoid pesticides;
(4) IATA case study using the DNT-IVB as a follow-up to the application of in silico models to screen compounds for potential DNT hazard identification.

This initial approach is considered sufficient to cover the main regulatory needs, i.e., screening and prioritization or hazard characterization, with the hope that additional IATA case studies will be developed to corroborate the use of the DNT-IVB. As such, the guidance should be considered as a living document able to include additional new case studies and/or testing methods that satisfy the readiness criteria and that represent a scientific step forward in the understanding of toxicity pathways in DNT. In addition, there is provision for guidance and case studies to be added to specifically include the zebrafish model as an alternative assay platform. The inclusion of the zebrafish model only partially addresses the underlying concept behind the alternative framework, as this model typically does not provide information on the mechanism of toxicity. However, it remains relevant and valuable because with the absence of data from mammalian models (e.g., rodents), it represents a strategic (i.e., rapid and inexpensive) and metabolically competent in vivo assay for which ethical and economical issues are perhaps less problematic as compared to rodent-based assays.

### 4.2. Adverse Outcome Pathways

The AOP conceptual framework describes a sequence of measurable key events (KEs) which are expected to be triggered by the activation of a molecular initiating event, MIE. This cascade of KEs finally results in an adverse outcome (AO) [46,47] of regulatory relevance. The adverse outcome pathway (AOP) concept would therefore facilitate the application of mechanistic knowledge to regulatory decisions. The AOPs also represent the ideal tool to identify experimental data gaps or lack of knowledge in the pathophysiological process. Indeed, the regulatory acceptance of NAMs, and particularly of DNT mechanistic endpoints which currently lack specific guidance, needs a strong biological rationale. The understanding of the pathological pathways leading from functional in vitro endpoints to behavioral changes at organism level appears particularly challenging. This is because adverse behavioral effects are difficult to measure (compared to, e.g., tumor/histopathology) and many variables may influence the experimental results in rodents. Moreover, for human real-world situations, many, e.g., genetic, environmental, social, modifiers may impact the outcome [6,13]. To reduce this uncertainty, an AOP-informed IATA offers a biological context to increase the scientific confidence for using mechanistic information in regulatory decision-making. This is because the AOP allows for a structured organization of evaluated information for which a biological causal relationship between the MIEs the downstream KEs and the AO can be documented, and a quantitative uncertainty assessment can be done [12].

The iterative approach in the IATA framework would therefore be a combination of building blocks where the AOP is paving the biological understanding for addressing the regulatory problem formulation. AOPs will assist in structuring the available information in terms of MIE, KEs and AO, and in the identification of missing data and/or in the design of testing strategies. One relevant consideration is that the endpoints measured in the DNT-IVB, in the AOP conceptual framework, represent KEs and not MIEs. However, more downstream KEs, closer to the AO, are expected to be more comprehensive in terms of coverage of potentially relevant pathways and the expected propagation of effects within the AOP to higher levels of organization may be more certain and consequently regulatory relevant.

It is therefore important to establish the amount of data to be gathered in an AOP-informed IATA, still based on problem formulation. More uncertainty can be an acceptable condition for screening and prioritization of a large number of chemicals. This might be less acceptable for, e.g., single chemical risk assessments. In this case, the DNT-IVB will be part of the mechanistic evidence influencing the overall WoE for the postulated AOP. The use of a prototype chemical stressor can be therefore an option to inform on the most probable MIE.

Mapping the available DNT-IVB data in the AOP wiki (https://aopkb.oecd.org/ and https://aopwiki.org/) enables the generation of DNT AOP networks and provides evidence that the DNT-IVB is indeed testing the fundamental neurodevelopment KEs in an AOP network [12]. This greatly facilitates the use of the DNT-IVB data for overall WoE in the IATA case studies, and the identification of missing data and/or design of testing strategies in accordance with the IATA iterative process. This approach will be described in the guidance through the case studies.

One critical limitation is that there are currently very few DNT AOPs that are not thyroid-related in the AOP Knowledgebase and AOP wiki (https://aopkb.oecd.org/ and https://aopwiki.org/) that have been reviewed and accepted through the OECD process. While missing fully characterized AOPs is an uncertainty, the increase in confidence for the regulatory use of the DNT-IVB may be based on the fact that it is covering multiple important neurodevelopmental processes (functional KEs). The AOP framework supporting regulatory decisions may therefore include putative AOP networks postulated using the outcome of the DNT-IVB. Indeed, where the amount of mechanistic information is scarce or lacking, the DNT-IVB will be the source for the identification of KEs that can be pragmatically framed in the AOP and used for the overall estimation of the regulatory weight of evidence.

### 4.3. Regulatory Engagement

The consensus statement [27] involved several stakeholders acknowledging that a more formal regulatory step is needed for the use of alternative methodologies for exploring and assessing the potential of a chemical to induce DNT. The OECD DNT expert group, and the development of guidance for the use and interpretation of the DNT-IVB, represents the summit of more than a decade of effort from academia and research institutions in the field of DNT. The framework therefore offers the opportunity to change the DNT testing paradigm in regulatory risk assessment. Multiple regulatory agencies and research institutions are at the helm of this process under the OECD umbrella (e.g., US EPA, NTP, Leibniz Institute for Environmental Medicine, University of Konstanz, Danish EPA, EFSA, European Union Joint Research Centre), but the involvement of additional institutions would be welcomed and should be encouraged. In the European context, for example, it will be strategically important to actively include additional agencies beyond the current institutional engagement in the process. This is important not only for the scientific benefit but also for the correct positioning of the DNT-IVB in the European jurisdiction of regulated chemicals with the engagement of the risk managers in defining the regulatory road map during and after publication of the guidance by the OECD. The strengthening of the regulatory engagement will encourage regulatory use of the DNT-IVB. This will also depend on whether specific data requirements for DNT assessment are adopted or changed by different jurisdictions, if a tiered approach for DNT is necessary and if specific test guidelines are written which encourage commercial laboratories to set up and run these assays.

## 5. Conclusions

One of the main duties of regulatory risk assessors dealing with environmental chemicals is to ensure that their use is not harmful to exposed populations. The consequences of neurodevelopmental disorders have a relevant socioeconomical impact and, while the recent increase in the prevalence of neurodevelopmental disorders has been associated with pre- and postnatal exposure to environmental chemicals, much remains unknown about causal relationships. DNT hazard identification remains a critical information gap that needs bridging to address these concerns. This information gap is due in part to the limited data requirements for DNT across the different jurisdictions and the uncertainties associated with the current in vivo assay-based DNT assessments [13]. This concern culminated in a consensus statement that a new framework for assessment of chemicals with the potential to disrupt brain development needs to be adopted [27].

It is therefore critical from the regulatory perspective to develop a framework based on NAMs in a regulatory context of international value, by adopting, as an underlying scientific principle, the assumption that dis-regulation of a fundamental process in brain development has the potential to lead to an unhealthy outcome.

The best option to resolve this was to first develop guidance on the use and interpretation of a DNT-IVB. This guidance should cover different regulatory problem formulations accompanied by a definition of the acceptable level of uncertainty for the DNT hazard characterization [18,25]. The OECD DNT expert group acknowledged that the transition from method development to test implementation needs to be achieved with a reasonably acceptable level of uncertainty, which is dependent on the regulatory problem formulation. Because of the high complexity of developmental neurotoxicity, multiple tests have been developed and assembled in a battery, for which readiness criteria were achieved.

Although multiple tests have been developed, the critical step of their comparison using larger chemical libraries is still lacking. Recently, this issue has been reduced by the publication of more experimental work testing a consolidated list of compounds in the DNT-IVB [28]. These considerations led the OECD DNT experts to propose a significant step in the regulatory application of the DNT-IVB, namely the decision to develop guidance on the use and interpretation of data from in vitro DNT assays (with the inclusion of the zebrafish model). The guidance will be strengthened by the inclusion of several IATA case studies based on regulatory problem formulations, and this would allow for the immediate implementation of the framework. The IATA represents the best tool for an iterative approach to answer the defined question in each specific regulatory context by considering the acceptable level of associated uncertainty.

## 6. Disclaimers

Andrea Terron is a staff member of the European Food Safety Authority (EFSA) and the opinions expressed are not necessarily reflect the official views of the EFSA.

The opinions expressed and arguments employed herein are those of the authors and do not necessarily reflect the official views of the OECD or of the governments of its member countries.

Preparation of this document has been funded in part by the U.S. Environmental Protection Agency. This document has been subjected to review by the US EPA’s Office of Research and Development and approved for publication. Approval does not signify that the contents reflect the views of the Agency, nor does mention of trade names or commercial products constitute endorsement or recommendation for use.

## Figures and Tables

**Table 1:** Summary of the DNT assays in the battery.

Neurodevelopmental Event	Cell Type	Endpoint	Site	Reference
Proliferation	hNP1	BrdU incorporation	USEPA	[29]
	Human Neuroprogenitor Cells (hNPCs) Grown in Neurospheres	BrdU incorporation; size	Düsseldorf	[30,31,32]
Migration	hiPSC-Derived Neural Crest Cells	Number of cells migrating into defined area of well.	Konstanz	[33]
	hNPCs Grown in Neurospheres	Migration of: Radial Glia Early neurons Oligodendrocytes	Düsseldorf	[34]
Differentiation	hNPCs Grown in Neurospheres	GFAP/NESTIN (radial glia) βIII tubulin (neurons) O4 (Oligodendrocytes)	Düsseldorf	[34]
Neurite Outgrowth	hN2/CDI I_gluta_	βIII tubulin incorporation	US EPA	[29,35,36]
	Rat Cortical	βIII tubulin incorporation	US EPA	[29,37]
	LUHMES	Neurite area visualized with calcein AM	Konstanz	[38]
	Human Dorsal Root Ganglion	Neurite area visualized with calcein AM	Konstanz	[39,40]
	hNPCs Grown in Neurospheres	βIII tubulin	Düsseldorf	[34]
Synaptogenesis	Rat Cortical Culture	Synaptic puncta (synaptophysin staining; HCI)	US EPA	[29,41]
Network Formation	Rat Cortical Culture	Electrical activity development measured by microelectrode array recordings	US EPA	[42,43]

## Data Availability

Not applicable.
